# Simultaneous Ablation of Ventricular Tachycardia and Hemodynamic Improvement of Mid-ventricular Obstructive Hypertrophic Cardiomyopathy by Coronary Venous Ethanol Ablation

**DOI:** 10.19102/icrm.2024.15062

**Published:** 2024-06-15

**Authors:** Serkan Topaloglu, Veysel Kutay Vurgun, Ahmet Korkmaz, Meryem Kara, Elif Hande Ozcan Cetin, Duygu Kocyigit Burunkaya, Firat Ozcan, Serkan Cay, Ozcan Ozeke, Sedat Kose, Miguel Valderrábano

**Affiliations:** 1 Department of Cardiology, Health Sciences University, Ankara City Hospital, Ankara, Turkey; 2 Department of Cardiology, Liv Hospital, Ankara, Turkey; 3 Houston Methodist DeBakey Heart and Vascular Center, Houston Methodist Hospital, Houston, TX, USA

**Keywords:** Coronary venous system, hypertrophic cardiomyopathy, mid-myocardial obstruction, retrograde alcohol, ventricular tachycardia

## Abstract

Transvenous coronary ethanol ablation may be successfully applied to simultaneously treat ventricular arrhythmia superimposed within a segment of hypertrophic cardiomyopathy. This presentation nicely describes this emerging technique for ventricular tachycardia ablation and identifies potential additional benefits of venous ethanol administration in patients with left ventricular obstructive physiology.

## Case presentation

A 51-year-old man with hypertrophic cardiomyopathy (HCM), mid-ventricular obstruction (MVO), and an apical aneurysm was referred to our center for stereotactic body radiation therapy (SBRT) after a protracted history of ventricular tachycardia (VT).

He had a transvenous implantable cardioverter-defibrillator (ICD) implanted in 2019 after syncopal events. Three years after ICD implantation, he was admitted with a refractory electrical storm. After failed oral treatments with propranolol, amiodarone, sotalol, and mexiletine, he underwent an endo-epicardial VT ablation in December 2022 at another tertiary center, in which a tachycardia with a cycle length (CL) of 350 ms was ablated using both bipolar ablation and half-normal saline (HNS) irrigation. However, 1 month later, his VT recurred slowly with a CL of 420 ms and did not respond to oral disopyramide therapy. Then, he was referred for a possible evaluation of SBRT.

**Figure 1:**
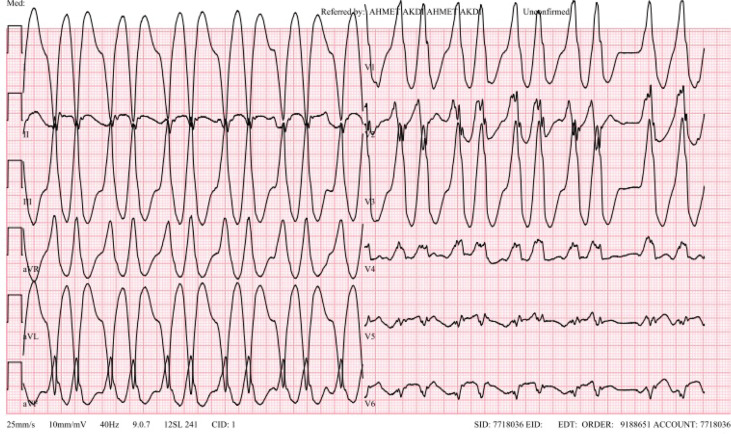
The morphology of the ventricular tachycardia is right bundle branch block and left-axis deviation.

His functional capacity was New York Heart Association (NYHA) class III. He had no family history of HCM. Echocardiography revealed HCM with an MVO and an apical aneurysm **(Videos 1 and 2)**. A hemodynamic assessment revealed a significant intraventricular pressure gradient of 35 mmHg. Despite the maximal dose of a β-blocker and amiodarone reloading, VT recurred. We proceeded with a repeat endo-epicardial VT ablation, which showed an apparent mid-lateral left ventricular (LV) focal activation pattern with simultaneous endo-epicardial timing, suggesting a mid-myocardial substrate **(Video 3)**. With two THERMOCOOL SMARTTOUCH™ SF catheters (Biosense Webster, Diamond Bar, CA, USA), we delivered sequential unipolar and bipolar endocardial and epicardial ablation at a power of 35 W, prolonged up to 3 min, using HNS irrigation. Despite this, the following day, VT recurred, both sustained and non-sustained and with the same morphology **(Figure 1)**, and remained nearly incessant for the following 2 weeks.

**Figure 2:**
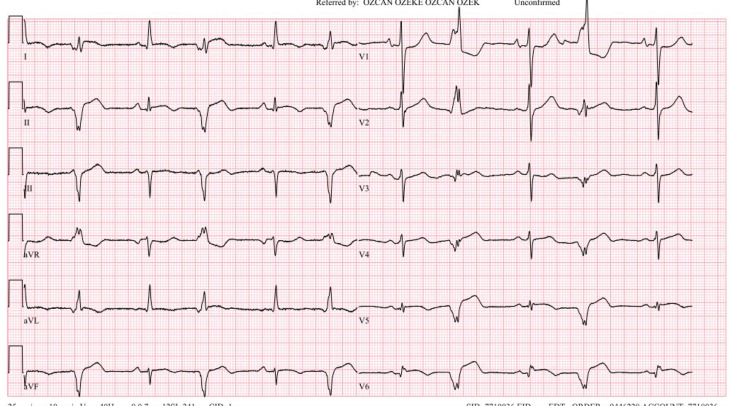
The following day, sustained ventricular tachycardia converted to bigeminal ventricular extrasystoles and finally totally normalized the subsequent day.

We then performed a venous ethanol ablation. A deflectable sheath (Agilis; Abbott, Chicago, IL, USA) was engaged in the coronary sinus. Venograms identified a lateral vein in the region of the mapped endo-epicardial exit area **(Video 4)**. The lateral vein was cannulated with a 0.0014″ floppy guidewire (Boston Scientific, Marlborough, MA, USA) and an angioplasty balloon (over-the-wire balloon; Boston Scientific). We first deployed a single-balloon technique in the lateral vein using a peripheral balloon distal to the lateral vein. Then, a smaller balloon (Gateway over-the-wire balloon; Boston Scientific) was placed in the proximal portion of the lateral vein **(Figure 2)**. Before injection of alcohol, the position of the balloon was verified by myocardial contrast echocardiography **(Video 5)**. Each vessel was occluded by injection of 5 mL of absolute alcohol in portions of 1 mL/min, and we applied a total of 45 mL of alcohol for ablation via the posterolateral branch **(Videos 6 and 7)** and the middle cardiac vein (MCV) targeting the apical aneurysm **(Video 8)**. The patient had slight chest pain during the procedure, and we detected a 1-mm inferolateral ST-segment elevation without coronary artery compromise. Programmed ventricular stimulation after ablation revealed non-inducibility. 

**Figure 3:**
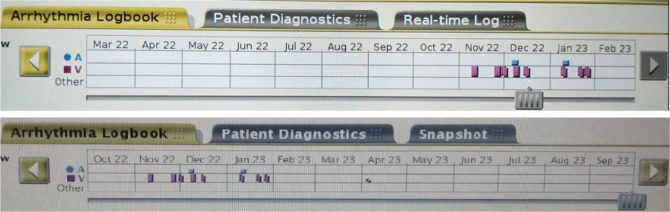
Device-interrogation data show implantable cardioverter-defibrillator therapy 1 year post-ablation.

**Figure 4:**
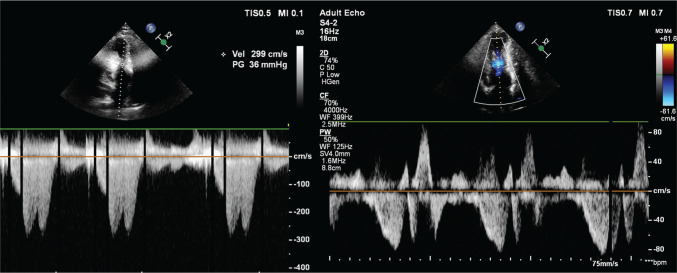
The control echocardiographic examination shows a reduction in the interventricular gradient from 36 to 6 mmHg.

**Figure 5:**
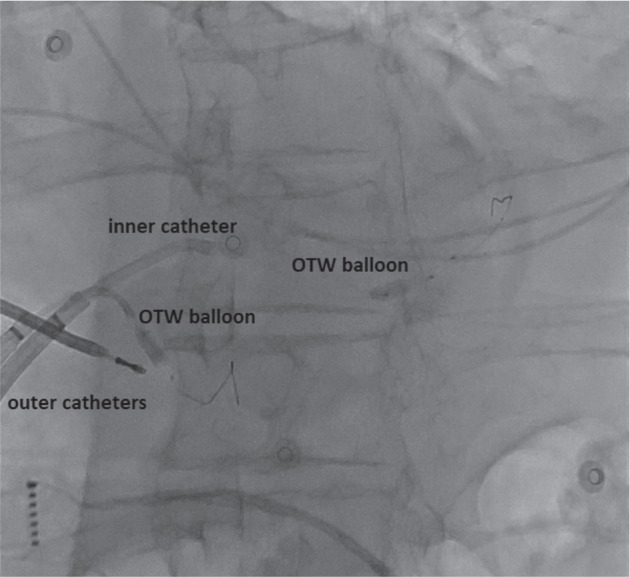
Ethanol ablation for the posterolateral branch of the coronary sinus. *Abbreviation:* OTW, over-the-wire.

The following day, he had bigeminal ventricular extrasystoles, which normalized the subsequent day **(Figure 3)**. The patient was discharged after an uneventful hospital stay of 5 days. Medication with 100 mg of metoprolol was continued at discharge without anti-arrhythmics. At an 11-month follow-up, the patient had no ICD shock or therapy, with only one non-sustained VT episode not requiring ICD therapy **(Figure 4)**. His functional capacity was dramatically improved (NYHA class II). The control echocardiographic examination showed a reduction in the interventricular gradient from 36 to 6 mmHg **(Figure 5)**, without apparent wall motion abnormalities **(Video 9)**.

## Discussion

HCM is a common inherited cardiac disease with a prevalence of approximately 0.2%–0.5% in the general population. The majority of HCM patients have a left ventricular outflow tract obstruction (LVOTO) at rest or with provocation.^1^ MVO is a less-common subtype of HCM, but it is associated with ventricular arrhythmia (VA) and a worse prognosis.^2–6^ Septal reduction therapy is recommended in patients with obstructive HCM who remain symptomatic under maximally tolerated optimal medical treatment.^7^ Alcohol septal ablation is a favorable option, especially in LVOTO-type patients with a high surgical risk or who refuse surgery.^8^ The procedure causes a controlled myocardial infarction of the basal portion of the interventricular septum by the injection of absolute alcohol to reduce LVOTO and improve the patient’s hemodynamics and symptoms.

Recently, numerous observations have demonstrated that the MVO type of HCM may also respond favorably to alcohol septal ablation.^9,10^ Fourth septal branch ablation for pressure gradient reduction in patients with the MVO type was reported by Seggewiss and Faber.^9^ In our case, the reduction in the intraventricular gradient was achieved with non-septal alcohol ablation. However, the effect of alcohol ablation of MCV and percutaneous endo-epicardial ablation might also have an impact on this favorable outcome.^11^

Catheter ablation is a useful option for patients with recurrent, drug-refractory, monomorphic VT, as well as device therapy. The success of catheter ablation depends on the ability to reach the anatomic location of the VT substrate. Indications for catheter ablation for VAs in patients with HCM are not clearly defined and need to be tailored for each patient.^12^ Santangeli and colleagues found that epicardial ablation was necessary to treat VT in close to 60% of cases.^13^ Therefore, understanding the location and extent of the substrate can help in guiding the ablation strategy. SBRT, sympathetic denervation, cardiac surgery, bipolar ablation, and alcohol ablation are promising therapies for VT refractory to catheter ablation. As seen in the current case, for some patients with focal ectopies arising from the epicardial or intramural locations, neither endocardial nor epicardial ablation is consistently helpful, as a result of the inadequate power delivery to the mid-myocardial VA origin.^14^ Venous ethanol is emerging as a powerful alternative in cases of unreachable substrate.^15^ Multiballoon, multivein intramural venous alcohol ablation can provide effective substrate ablation in patients with radiofrequency catheter ablation-refractory VT in the setting of structural heart disease over a broad range of LV locations.^16^ Despite a long duration, HNS, and bipolar ablation attempts, the only retrograde alcohol application of the posterolateral branch of the coronary sinus eliminated the patient’s nearly incessant tachycardia in the current case. Coronary arterial alcohol ablation could have achieved a greater reduction in mid-myocardial obstruction in addition to VT elimination; however, it is riskier than ablating the venous system. Therefore, ethanol ablation via the coronary venous anatomy can offer electrophysiologists another option in their armamentarium of treatments against VT.

## Figures and Tables

**Video 1:** Modified parasternal long-axis view shows midventricular obstruction.

**Video 2:** Apical four-chamber view shows to and fro flow at the midventricular level.

**Video 3:** Activation mapping of VT shows simultaneous endo-epicardial exit suggesting an intramural circuit at the lateral LV wall.

**Video 4:** Coronary venous angiogram showing posterolateral branch.

**Video 5:** S Injection of echocardiographic saline contrast into the target lateral branch opacifies the mid part of the lateral wall, verifying the optimal choice of septal branch.

**Video 6:** Targeting lateral branch of coronary sinüs by long balloon occlusion.

**Video 7:** Targeting lateral branch of coronary sinüs by short balloon occlusion.

**Video 8:** Targeting lateral branch of coronary sinüs by middle cardiac vein.

**Video 9:** Post ablation echocardiography shows no apparent wall motion abnormality or contractile dysfunction.
